# Multimodal Monitoring of Cardiovascular Responses to Postural Changes

**DOI:** 10.3389/fphys.2020.00168

**Published:** 2020-03-03

**Authors:** Arjen Mol, Andrea B. Maier, Richard J. A. van Wezel, Carel G. M. Meskers

**Affiliations:** ^1^Department of Human Movement Sciences @AgeAmsterdam, Amsterdam Movement Sciences, Vrije Universiteit Amsterdam, Amsterdam, Netherlands; ^2^Department of Biophysics, Donders Institute for Brain, Cognition and Behaviour, Radboud University, Nijmegen, Netherlands; ^3^Department of Medicine and Aged Care @AgeMelbourne, The Royal Melbourne Hospital, The University of Melbourne, Parkville, VIC, Australia; ^4^Department of Biomedical Signals and Systems, Technical Medical Centre, University of Twente, Enschede, Netherlands; ^5^Department of Rehabilitation Medicine, Amsterdam UMC, Amsterdam Movement Sciences, Vrije Universiteit, Amsterdam, Netherlands

**Keywords:** baroreceptor reflex, cerebral autoregulation, electrocardiography, near-infrared spectroscopy, orthostatic hypotension

## Abstract

**Background:**

In the poorly understood relationship between orthostatic hypotension and falls, next to blood pressure (BP), baroreflex sensitivity (BRS) and cerebral autoregulation (CAR) may be key measures. The posture- and movement dependency of orthostatic hypotension requires continuous and unobtrusive monitoring. This may be possible using simultaneous photoplethysmography (PPG), electrocardiography (ECG), and near-infrared spectroscopy (NIRS) signal recordings, from which pulse wave velocity (PWV; potentially useful for BP estimation), BRS and CAR can be derived. The PPG, NIRS and PWV signal correlation with BP and BRS/CAR reliability and validity need to be addressed.

**Methods:**

In 34 healthy adults (mean age 25 years, inter quartile range 22–45; 10 female), wrist and finger PPG, ECG, bifrontal NIRS (oxygenated and deoxygenated hemoglobin) and continuous BP were recorded during sit to stand and supine to stand movements. Sixteen participants performed slow and rapid supine to stand movements; eighteen other participants performed a 1-min squat movement. Pulse wave velocity (PWV) was defined as the inverse of the ECG R-peak to PPG pulse delay; PPG, NIRS and PWV signal correlation with BP as their Pearson correlations with mean arterial pressure (MAP) within 30 s after the postural changes; BRS as inter beat interval drop divided by systolic BP (SBP) drop during the postural changes; CAR as oxygenated hemoglobin drop divided by MAP drop. BRS and CAR were separately computed using measured and estimated (linear regression) BP. BRS/CAR reliability was defined by the intra class correlation between repeats of the same postural change; validity as the Pearson correlation between BRS/CAR values based on measured and estimated BP.

**Results:**

The highest correlation with MAP was found for finger PPG and oxygenated hemoglobin, ranging from 0.75–0.79 (sit to stand), 0.66–0.88 (supine to stand), and 0.82–0.94 (1-min squat). BRS and CAR reliability was highest during the different supine to stand movements, ranging from 0.17 – 0.49 (BRS) and 0.42-0.75 (CAR); validity was highest during rapid supine to stand movements, 0.54 and 0.79 respectively.

**Conclusion:**

PPG-ECG-NIRS recordings showed high correlation with BP and enabled computation of reliable and valid BRS and CAR estimates, suggesting their potential for continuous unobtrusive monitoring of orthostatic hypotension key measures.

## Introduction

Orthostatic hypotension (OH), defined as a blood pressure (BP) drop of at least 20 mm Hg systolic and/or >10 mm Hg diastolic after postural change (Freeman et al., 2011), is highly prevalent in older adults (Cooke et al., 2013; Frewen et al., 2014; Timmermans et al., 2018), whereas the effectiveness of non-pharmacological interventions are limited (De Bruïne et al., 2017). OH may be accompanied by clinical symptoms, e.g., lightheadedness and dizziness (Juraschek et al., 2017, 2018), and is associated with poor clinical outcome such as impaired physical performance (Pasma et al., 2014; de Bruïne et al., 2018; Mol et al., 2018b), falls (Saedon et al., 2016; Mol et al., 2018a), cognition (Iseli et al., 2019), cardiovascular diseases (Verwoert et al., 2008; Ricci et al., 2015) and mortality (Verwoert et al., 2008; Lagro et al., 2012; Rockwood et al., 2012; Ricci et al., 2015; Frith et al., 2016). Sensitivity for the diagnosis of OH is higher for continuous BP measurement than for intermittent BP measurements after standing up, and continuous BP measurement has shown to be stronger associated with physical performance (Pasma et al., 2014; de Bruïne et al., 2018). However, clinical orthostatic BP measurements do not account for many of the symptoms and falls patients experience at home, due to the time varying and posture- and movement dependent nature of orthostatic BP drop, resulting in a poor reproducibility of the OH diagnosis (Frith, 2015). Furthermore, the baroreflex (i.e., change in interval between heart beats as a response to BP changes) and cerebral autoregulation (CAR, i.e., regulation of cerebral blood flow during BP changes) are mechanisms that potentially attenuate the clinical consequences of OH and are therefore essential to understand the relationship between OH and clinical outcome (James and Potter, 1999; Mehagnoul-Schipper et al., 2000; Saint Martin et al., 2013; Tarumi et al., 2014; Ziegler, 2018). BRS and CAR are not addressed during regular clinical BP measurements (Mol et al., 2018c; Zanotto et al., 2018). There is therefore a need for continuous, unobtrusive, simultaneous assessment of orthostatic BP, baroreflex sensitivity (BRS) and CAR, which cannot be performed using the devices currently used in clinical practice.

Elucidation of the relationship between OH and clinical outcome (i.e., physical performance, cognitive performance and falls) through continuous assessment of BP, BRS and CAR in the home situation may be possible using a combination of non-invasive measurements, encompassing photoplethysmography (PPG), ECG and near-infrared spectroscopy (NIRS). PPG may enable monitoring BP in superficial arteries, e.g., the radial artery (wrist) or digital artery (finger). PPG amplitude was reported to correlate with BP (Hennig and Patzak, 2013; Lim et al., 2015; Gao et al., 2016; Sun et al., 2016; Ding et al., 2017; Wang et al., 2018). When combined with ECG, PPG can be used to compute pulse wave velocity (PWV), a parameter reflecting both BP, arterial stiffness and arterial vasoconstriction (Salvi, 2017). To assess CAR, cerebral oxygenation measured using NIRS may be used as a proxy for cerebral blood flow (Steiner et al., 2009; Kainerstorfer et al., 2015; Mol et al., 2019).

Prerequisites for clinical application of the BP, BRS and CAR monitor during postural changes are (a) correlation of PPG, NIRS and derived PWV with BP after postural change, to enable BP estimation, (b) good reliability and validity of BRS/CAR estimates and (c) evidence for the potential additional value of BRS estimates assessed during postural change compared to conventional validated BRS measures assessed in rest. In the present proof-of-concept study in a cohort of healthy adults we will address these prerequisites during different types and speeds of postural changes by calculating the correlations between PPG, NIRS and PWV signals, and measured BP; intra class correlation between repeats of postural changes; correlations between BRS/CAR estimates based on estimated and measured BP; and correlations between BRS assessed during postural change and in rest.

## Materials and Methods

Thirty-four participants were recruited by oral and written advertisement in a university teaching setting at the Radboud University in Nijmegen, The Netherlands. Sixteen participants were primarily recruited from university students (subgroup 1), while 18 participants were primarily recruited from university employees (subgroup 2). Participants were included if they were younger than 65 years, and had no history of cardiovascular, respiratory or neurological disorders resulting in impaired functioning.

### Ethics Statement

The study was performed in accordance with the Declaration of Helsinki and approved by the Ethics Committee of the Faculty of Science of the Radboud University in Nijmegen. All participants signed informed consent.

### Participant Characteristics

Information about age, height, weight, handedness, alcohol usage, smoking habits and medication use was obtained from all participants.

### Instrumentation

Two PPG sensors were customized to measure synchronized output signals with a sampling frequency of 1000 Hz and were applied to the left radial artery (wrist) and digital artery (distal digital finger). The left arm was placed in a sling to minimize differences in height between the PPG measurement location and the heart. A digital tilt meter was attached to the participants’ trunk to measure the angle relative to the horizontal plane and to identify the start of postural change. Data recording was performed using a customized application developed in MATLAB R2017b (MathWorks, Natick, MA, United States).

Two PortaLite NIRS sensors (Artinis Medical Systems B.V., Elst, Netherlands; sampling frequency of 50 Hz) were applied bilaterally to the forehead, approximately 2 cm above the eyebrows. Differential pathway factors were estimated based on wavelength and age (Scholkmann and Wolf, 2013). Oxygenated hemoglobin (O_2_Hb) and deoxygenated hemoglobin (HHb) were computed using the modified Lambert-Beer law (Kocsis et al., 2006). Data recording was performed using Oyxsoft v3 (Artinis Medical Systems B.V., Elst, Netherlands).

A 5-lead ECG and continuous BP and was measured using a Finapres non-invasive hemodynamics monitor (Finapres NOVA, Finapres Medical Systems, Amsterdam, Netherlands), and applied to the left middle finger. This monitor includes a module measuring the height of the finger relative to the heart to enable reconstruction of BP at heart level from BP at finger level.

A common analog reference signal was imported into all devices to enable off-line synchronization of the signals. The reference signal consisted of a train of 16 pulses for each minute, each pulse train coding the time from the start of the experiment in minutes. Off-line synchronization and storage, and further analysis of the signals was performed using MATLAB R2017b (MathWorks, Natick, MA, United States).

### Protocol

Participants were asked to void urine before start of the experiment. Room temperature was kept between 20 and 23 °C. Participants were stimulated to relax, instructed not to talk and to limit movements not related to tasks.

**FIGURE 1:**
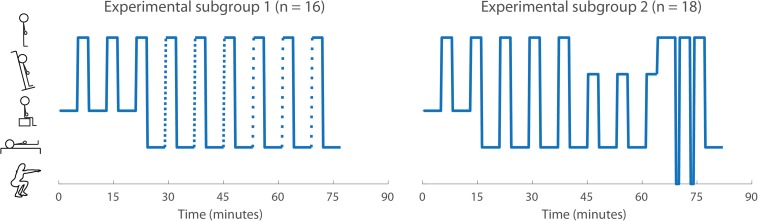
The experimental protocol for both subgroups (adapted from Mol et al., 2019). The symbols on the *y*-axis indicate (from top to bottom): active standing, head-up tilt, sitting, supine, and squat position. Transitions shown as solid lines indicate preferred speed and dashed lines with small and wide gaps indicate rapid and slow transitions, respectively.

To keep the total measurement duration per participant within 2 h, each of the two recruited subgroups underwent a different experimental protocol (Figure 1). Subgroup 1 performed the following postural changes: A (sit to stand, i.e., standing up from sitting position at the preferred speed of the individuals), B (slow supine to stand, i.e., standing up from supine position in approximately 10 s) and C (rapid supine to stand, i.e., standing up from supine position in approximately 3 s). Subgroup 2 performed postural changes A, D (supine to stand at preferred speed), E (head up tilt, i.e., tilting from supine position to 70-degree tilt in 15 s without use of leg muscles) and F (1-min squat, i.e., an isometric leg exercise test increasing BP). Postural changes A-E were preceded by a 5-min resting period [to reach steady state of BP regulation (Frith, 2015)] and followed by a 3-min standing period.

All postural changes were performed in blocks of three repeats per block. Only two repeats per block were performed for postural change A and F in subgroup 2. The sequence of the blocks was randomized to prevent structural influences from preceding postural changes on following postural changes, except for the 1-min squat blocks, which were performed at the end of the protocol as these postural changes might induce fatigue.

### Signal Quality Assessment

PPG, NIRS and BP signals were inspected visually for each repeat of each postural change. Signals not showing a heartbeat for more than 10 s during baseline (i.e., the 60 s before testing), more than 10 s in the first minute after the start of the test or more than 20 s in minute two and three after the start of the test, were discarded.

### Data Preprocessing

PPG signals were filtered using a 0.05–10 Hz Butterworth band pass filter to compute PWV (Elgendi, 2012; Akdemir Akar et al., 2013; Sun et al., 2016). The PPG, NIRS and BP signals were resampled at 25 Hz. From these resampled signals, a standardized version (i.e., subtraction of baseline mean and division by baseline standard deviation) and a filtered version (using a 5-s moving average filter) were computed.

### Pulse Wave Velocity (PWV) Computation

Beat-to-beat PWV was computed using the ECG and the PPG signals and defined as the inverse of the time between the R-peak in the ECG to the peak in the first derivative of the PPG signal. Detection of peaks in the first derivative in the PPG signal was performed in two steps, to avoid detecting peaks not corresponding to the upstroke of the PPG wave on the one hand (i.e., a low specificity) and detecting no peaks at all (i.e., a low sensitivity) on the other hand. In the first step, a high specificity, low sensitivity PWV signal was computed, using high peak detection thresholds (i.e., 3 standard deviations of the surrounding 5 s of PPG signal). In the second step, a high sensitivity, low specificity PWV signal was computed using low peak detection thresholds (i.e., 1.5 standard deviations of the surrounding 5 s of PPG signal). To compute PWV for as many heartbeats as possible while preventing erroneous PWV calculation, the PWV values in the high sensitivity, low specificity signal exceeding the mean −3 SD or +5 SD of the high specificity, low sensitivity signals were discarded and the remaining signal was used for further analysis.

### Signal Correlation With BP

PPG, NIRS, and PWV signal correlation with BP was defined as their correlation with mean arterial pressure (MAP) within 30 s after each postural change. Filtered signals (5 s moving average window) were used as these were reported to show the most clinically relevant representation of the BP data (van der Velde et al., 2007). The signals were averaged over repeats.

### Baroreflex Sensitivity (BRS) and Cerebral Autoregulation (CAR)

BRS was defined as inter beat interval (IBI) drop divided by systolic BP (SBP) drop within 1 min after postural change. CAR was defined as O_2_Hb drop divided by MAP drop within 1 min after postural change. All computations were performed using the 5-s moving average filtered signals. BRS and CAR values exceeding five times the standard deviation of the other measurements of the same postural change were discarded.

**FIGURE 2:**
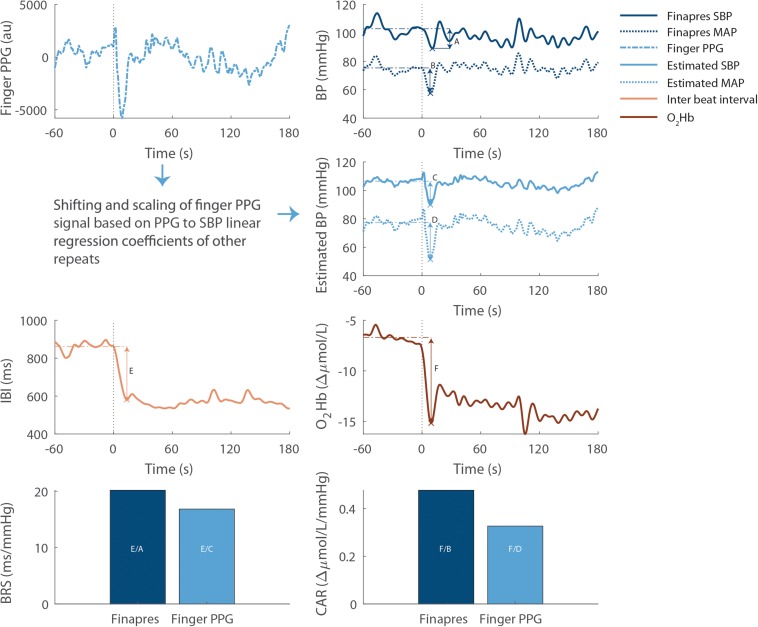
Example computation of baroreflex sensitivity and cerebral autoregulation for the first repeat of supine to stand at preferred speed in one participant. All signals are filtered using a 5-s moving average filter. The vertical dotted lines indicate the start of postural change, the horizontal dashed lines indicate baseline values and the crosses indicate the lowest value after standing up. As indicated in the bottom panels, baroreflex sensitivity is computed as inter beat interval drop (E) divided by the measured (A) or estimated (C) SBP drop. Similarly, cerebral autoregulation is computed as the cerebral oxygenation drop (F) divided by the measured (B) or estimated (D) MAP drop. PPG, photoplethysmography; au, arbitrary units; SBP, systolic blood pressure; IBI, inter beat interval; O_2_Hb, cerebral oxygenated hemoglobin. BRS, baroreflex sensitivity; CAR, cerebral autoregulation.

BRS and CAR were separately computed using measured BP and estimated BP (Figure 2). BP (both SBP and MAP) estimation was based on the finger PPG and performed for each participant, postural change (except the 1-min squat) and repeat, by using linear regression models with the PPG and BP signals in the interval between 0 and 30 s as independent and dependent variables, respectively. For a given repeat, PPG and BP signals of the other available repeats of the same subject and postural change were used to compute the regression coefficients. BP was then estimated as: *BP* = β_0_ + β_1_*PPG.

The following conventional validated BRS measures were assessed in rest: sequence method BRS and baroreflex effectiveness index (BEI) (Bertinieri et al., 1988; Di Rienzo et al., 2001; Laude et al., 2008; Silva et al., 2019). Sequence method BRS and BEI were computed based on the 5-min resting epochs preceding the sit to stand movements, using the criteria reported by Silva et al. (2019).

### Statistical Analysis

Normally distributed continuous variables were presented using a mean and standard deviation. Variables following other distributions were presented using the median and inter quartile range.

Signal correlation with BP was expressed using Pearson correlation coefficients.

BRS and CAR reliability was defined as their two-way mixed absolute single measure intra class correlation (ICC) between repeats of the same postural change. ICCs between 0 – 0.40, 0.40 – 0.59, 0.60 – 0.74, and 0.75 – 1 were regarded as poor, fair, good and excellent, respectively (Cicchetti, 1994).

BRS and CAR validity was defined as the Pearson correlation between BRS/CAR estimates based on measured and estimated BP.

The potential additional value of BRS estimates assessed during postural change compared to conventional validated BRS measures assessed in rest was expressed using their Pearson correlations, lower correlations indicating higher potential additional value.

## Results

Table 1 lists the participant characteristics. The median age of the 34 included individuals was 25 years [inter quartile range (IQR) 22–45; 10 female]. Median age of subgroup 1 and 2 was 22.5 years (IQR 21–24) and 37.5 years (26.5–56), respectively, and the number of included female individuals was 4 and 6.

**TABLE 1:** Participant characteristics, stratified by subgroups.

**Characteristic**	***N***	**All (*n* = 34)**	**Subgroup 1 (*n* = 16)**	**Subgroup 2 (*n* = 18)**
Age, years, median [IQR]	34	25 [22–45]	22.5 [21–24]	37.5 [26.5–56.0]
Male, n (%)	34	24 (70.6)	12 (75.0)	12 (66.7)
Height, m, median [IQR]	34	1.80 [1.72–1.85]	1.80 [1.75–1.86]	1.80 [1.67–1.85]
Weight, kg, median [IQR]	34	70.5 [65.8–75.0]	70.5 [67.3–74.8]	69.0 [63.8–76.5]
Right-handed, n (%)	34	29 (85.3)	16 (100)	13 (72.2)
Current smoking, n (%)	34	2 (5.9)	1 (6.3)	1 (5.5)
Excessive alcohol use, n (%)*	34	0 (0)	0 (0)	0 (0)
Medication use, n (%)	34	8^†^ (23.5)	3 (18.8)	5 (27.7)
Resting HR, bpm, median [IQR]	34	71 [66 – 78]	71 [66–77]	71 [66–79]
Resting SBP, mmHg, median [IQR]	34	130 [123 – 140]	126 [122–140]	131 [126–145]
Resting DBP, mmHg, median [IQR]	34	82 [76–87]	79 [73–85]	83 [78–96]
Time needed for sit to stand, s, median [IQR]	34	7.0 [5.2–8.0]	6.6 [5.3–9.3]	7.0 [5.2–8.0]
Time needed for slow supine to stand, s, median [IQR]	16	15.6 [14.1–19.1]	15.6 [14.1–19.1]	NA
Time needed for rapid supine to stand, s, median [IQR]	16	6.7 [5.7–8.7]	6.7 [5.7–8.7]	NA
Time needed for supine to stand at preferred speed, s, median [IQR]	18	9.0 [6.8–11.4]	NA	9.0 [6.8–11.4]
Time needed for head up tilt, s, median [IQR]	18	16.4 [16.1–17.4]	NA	16.4 [16.1–17.4]

Resting HR, SBP, and DBP were computed as the baseline mean. IQR, interquartile range; SD, standard deviation; BMI, Body Mass Index; HR, Heart rate; bpm, beats per minute; SBP, systolic blood pressure; DBP, diastolic blood pressure. *Excessive alcohol use is defined as >14 units per week for females and >21 units per week for males. ^†^Methylphenidate for attention deficit hyperactivity disorder (2 participants), terbutaline for asthma (2 participants), candesartan or hydrochlorothiazide for hypertension (2 participants), escitalopram for a mood disorder (1 participant), desloratadine for pollen allergy (1 participant).

### Signals

#### Signal Quality Assessment

After signal quality assessment, at least one repeat for each postural change was available for 31/34 subjects. The proportion of repeats showing good quality data was overall 329/355 (93%), and ranged among postural changes from 31/36 (86%; 1-min squat) to 57/59 (97%; head up tilt). The proportion of signals discarded after visual quality inspection was 8.5% (wrist PPG), 4.9% (finger PPG), 1.3% (NIRS), and 0.6% (BP). Tables 2, 3 show the available number of repeats per postural change and the number of repeats per signal discarded after data quality assessment, respectively.

**TABLE 2:** Data availability, repeats.

	**# Participants**	**# Repeats (# participants)**	Repeats discarded*, # repeats (# participants)
Sit to stand	34	3 (16), 2 (18)	6 (5)
Slow supine to stand	16	3	5 (4)
Supine to stand at preferred speed	18	3	4 (3)
Rapid supine to stand	16	3	4 (4)
Head up tilt	18	3	2 (1)
1-min squat	18	2	5 (3)

Data availability. Number of participants, number of repeats per participant and availability of extra repeat performed by second observer. *Repeats discarded due to technical problems affecting all signals such as problems with data storage or loss of the reference signal needed for proper synchronization of the signals.

**TABLE 3:** Data availability, signals.

	**Wrist PPG**	**Finger PPG**	**NIRS**	**BP**
Sit to stand	8 (5)	4 (3)	0	0
Slow supine to stand	10 (5)	5 (2)	0	1 (1)
Supine to stand at preferred speed	1 (1)	2 (2)	0	0
Rapid supine to stand	8 (6)	3 (2)	2 (1)	1 (1)
Head up tilt	0	0	0	0
1-min squat	1 (1)	2 (2)	2 (2)	0

*The table lists the number of discarded repeats per signal, stratified by test condition. The values in parentheses indicates the number of participants for whom one or more repeats per signals were discarded.*

#### Signal Characteristics

**FIGURE 3:**
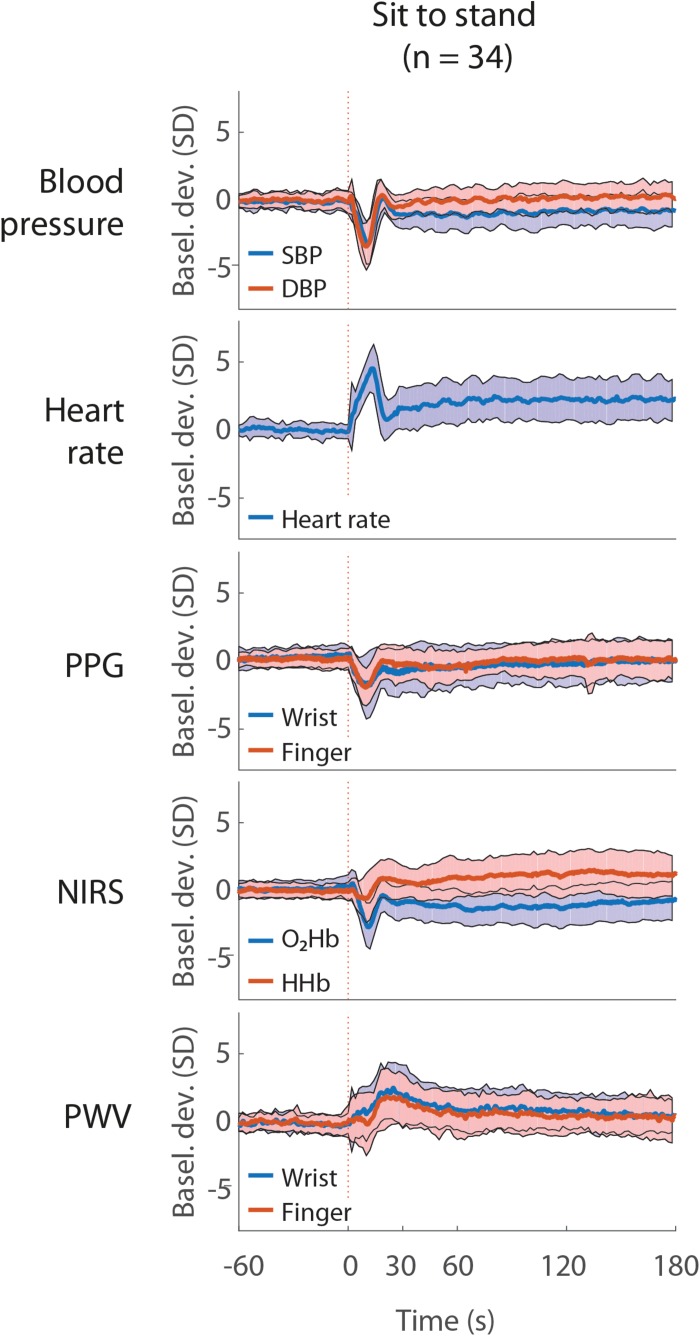
Average blood pressure, heart rate, photoplethysmography (PPG), near infrared spectroscopy (NIRS) and pulse wave velocity (PWV) during the sit to stand movement. All signals are unfiltered and normalized at baseline. The red vertical line indicates the onset of the postural change. The shaded areas indicate the standard deviation. The data represent 24 male subjects and 10 female subjects. Basel. dev., signal deviation from mean baseline; SBP, systolic blood pressure; DBP, diastolic blood pressure; O_2_Hb, oxygenated hemoglobin; HHb, deoxygenated hemoglobin.

Figure 3 shows the averaged signals during the sit to stand movement and Supplementary Appendix A additionally shows the responses to the supine to stand and head up tilt movements. The signals showed a similar response to sit to stand and supine to stand, and consisted of a temporary drop (BP, PPG, O_2_Hb, and HHb) or increase (heart rate and PWV) within 30 s after standing up, reaching a steady state at 60 s after standing up. The responses to head up tilt within 30 s were smaller than the responses to sit to stand and supine to stand movements.

**FIGURE 4:**
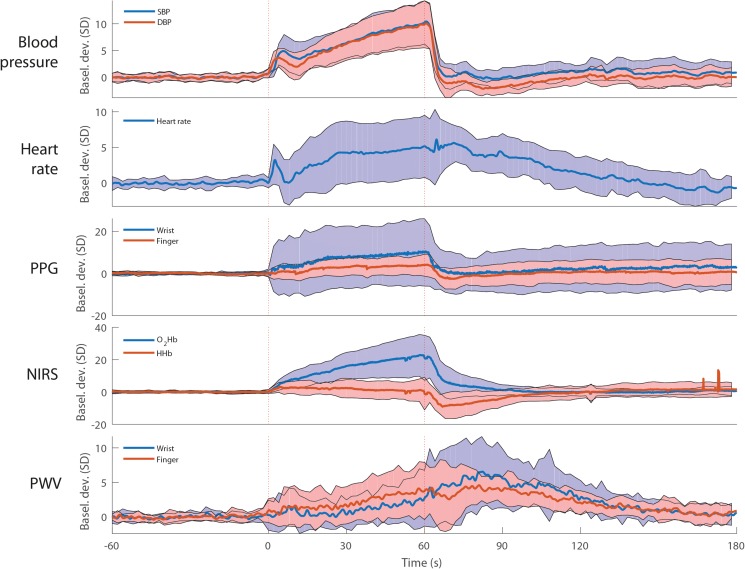
Average blood pressure, heart rate, photoplethysmography (PPG), near infrared spectroscopy (NIRS) and pulse wave velocity (PWV) during 1-min squat movement. All signals are unfiltered and standardized at baseline. The red dotted vertical lines indicate the onset and end of 1-min squat movement. The shaded areas indicate the standard deviation. The data represent 12 male subjects and 6 female subjects. *N* = 18. O_2_Hb, oxygenated hemoglobin; HHb, deoxygenated hemoglobin.

Figure 4 shows the averaged signals during and after a 1-min squat movement. All signals, except HHb showed an increase during squat. After standing up from squat position, wrist and finger PPG, O_2_Hb and HHb and BP showed a sudden drop, heart rate declined slowly and PWV signals increased to rise, reaching a peak at 20–30 s after standing up and declining thereafter. Signal characteristics (means, minima and maxima) after postural changes and during and after the 1-min squat movement are listed in Supplementary Appendices B, C.

### Signal Correlation With BP

**FIGURE 5:**
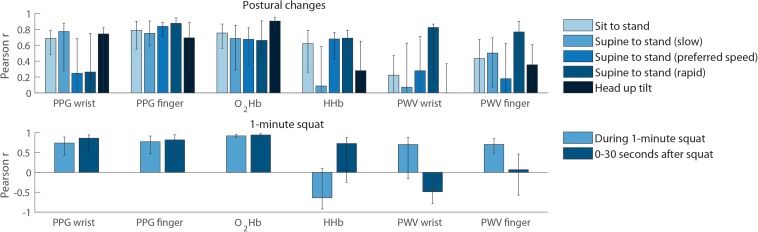
Signal correlation with blood pressure. **Top panel:** correlation with mean arterial pressure, evaluated over the first 30 s after postural change. All bars indicate the median and error bars indicate the inter quartile range. The data represent 24 male subjects and 10 female subjects. *N* = 34 (sit to stand), *n* = 16 (slow and rapid supine to stand) and *n* = 18 (supine to stand at preferred pace and head up tilt). **Bottom panel:** correlation with mean arterial pressure during and after the 1-min squat movement. The data represent 12 male subjects and 6 female subjects (*N* = 18). HHb, deoxygenated hemoglobin; MAP, mean arterial pressure; O_2_Hb, oxygenated hemoglobin; SBP, systolic blood pressure; SD, standard deviation; PPG, photoplethysmography; PWV, pulse wave velocity.

Figure 5 shows the PPG, NIRS and PWV signal correlation with BP after postural change. Finger PPG and oxygenated hemoglobin signals showed highest signal correlation with BP, ranging from 0.75–0.79 (sit to stand), 0.66–0.88 (supine to stand), and 0.82–0.94 (1-min squat).

### Baroreflex Sensitivity and Cerebral Autoregulation

#### Reliability and Validity

**FIGURE 6:**
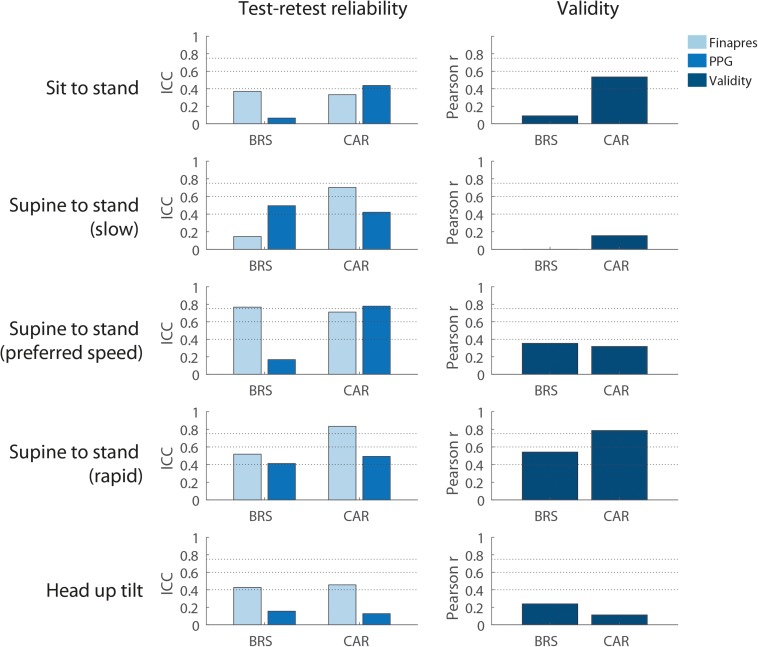
Baroreflex sensitivity and cerebral autoregulation. Reliability and validity for different postural changes. Reliability is shown for baroreflex sensitivity and cerebral autoregulation as assessed using the measured and estimated blood pressure. Rapid supine to stand gives most valid measurements of baroreflex and cerebral autoregulation. The data represent 24 male subjects and 10 female subjects. *N* = 34 (sit to stand), *n* = 16 (slow and rapid supine to stand), and *n* = 18 (supine to stand at preferred pace and head up tilt). BRS, baroreflex sensitivity; CAR, cerebral autoregulation; PPG, finger photoplethysmography.

Figure 6 shows BRS and CAR reliability and validity. BRS and CAR showed the highest ICC between repeats during supine to stand movements, ranging from 0.17 – 0.49 (BRS) and 0.42-0.75 (CAR). Correlation between BRS/CAR estimates based on measured and estimated BP was highest during rapid supine to stand movements, 0.54 and 0.79 respectively.

#### Potential Additional Value of BRS Estimates Assessed During Postural Change

**FIGURE 7:**
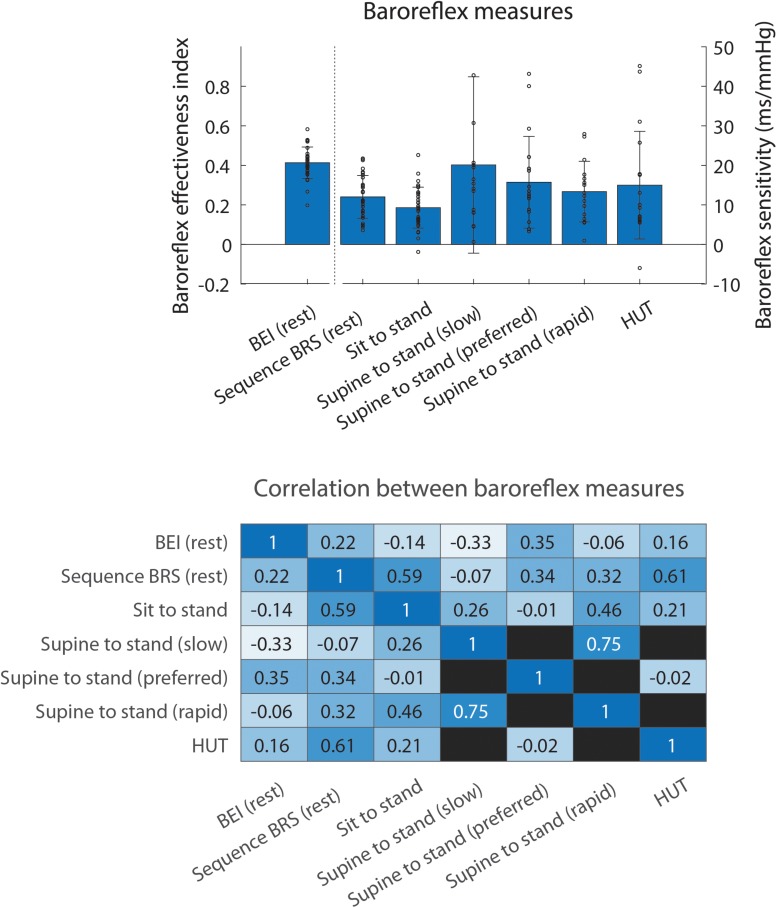
Comparison between baroreflex measures. Bars in the **top panel** show the mean of each baroreflex measure, error bars indicating the standard deviation and small circles the data points corresponding to individual subjects. The **bottom panel** shows the Pearson correlation coefficient of the correlation between the different measures. Empty squares indicate that the two baroreflex measures were not available for the same individuals. BEI, baroreflex effectiveness index; BRS, baroreflex sensitivity; HUT, head up tilt.

Figure 7 shows the comparison between BRS estimates assessed during postural change and conventional validated BRS measures assessed in rest. BRS assessed during sit to stand and head up tilt was correlated with sequence BRS (*r* = 0.59 and 0.61, respectively). Correlations between supine to stand movements and sequence BRS in rest were low (*r* = −0.07 – 0.34). All baroreflex measures showed a low correlation with baroreflex effectiveness index (*r* = −0.33 – 0.35).

## Discussion

In this proof-of-concept study in a cohort of healthy adults we found that combined PPG, ECG and NIRS signal recordings to estimate BP, baroreflex sensitivity (BRS) and cerebral autoregulation (CAR) during various postural changes showed high signal correlations with measured BP, particularly for finger PPG and NIRS derived oxygenated hemoglobin (correlations ranging from 0.66 to 0.94). Furthermore, we found that BRS was of poor to fair reliability and CAR was of fair to excellent reliability during the supine to stand movements. Correlations between BRS/CAR estimates based on estimated and measured BP were 0.54 and 0.79, respectively. Correlations between BRS estimates assessed during postural change and conventional validated BRS measures assessed in rest were particularly low for supine to stand movements, indicating the potential additional value of the BRS estimates assessed during these postural changes. These results suggest the potential clinical value of these techniques for continuous and unobtrusive monitoring of BP, BRS and CAR as key measures of OH.

To the best of our knowledge, this is the first study proposing and assessing a non-invasive technique that might be used for continuous monitoring of posture-related BP, BRS and CAR in patients with OH. Results on sensitivity and reliability of NIRS parameters in a subpopulation of this study (*n* = 15) were published before (Mol et al., 2019). Other previous research focused on specific aspects, such as PPG-based BP estimation (Chen et al., 2012; Sun et al., 2016), cerebral oxygenation changes during standing up (Mehagnoul-Schipper et al., 2000, 2001; van Lieshout et al., 2001) and posture related PWV changes (Foo and Lim, 2006), but never assessed a combined ambulatory technique for BRS and CAR monitoring.

### Signal Correlation With BP

The high correlation with BP found for finger PPG indicates that this signal might be used for continuous BP estimation during postural change. The high correlation of O_2_Hb with BP was not expected, as O_2_Hb is not only determined by BP, but also by CAR (Steiner et al., 2009; Kainerstorfer et al., 2015), which may act as a high-pass filter (Tarumi and Zhang, 2018), and cerebral microcirculation (Krakow et al., 2000). The results of the present study indicate that BP is a relatively large contributor to cerebral oxygenation.

Finger PWV correlated well with BP during the rapid supine to stand movement, but not during other postural changes, potentially due to the fact that BP drop is largest during rapid supine to stand. However, PWV in theory also reflects arterial stiffness and arterial vasoconstriction, which are of interest in patients with OH (Salvi, 2017). The association of PWV with arterial stiffness and arterial vasoconstriction was not addressed in the present study, but should be investigated in future studies by assessing the association of PWV with carotid intima-media thickness, as a measure of vessel stiffness (Koivistoinen et al., 2007; Torjesen et al., 2017; Ikonomidis et al., 2019), and by measuring PWV during hand grip exercise, which influences vascular sympathetic outflow and thereby vasoconstriction (Kamiya et al., 2001; Jarvis et al., 2011). If PWV can be demonstrated to be a good measure of vessel stiffness, it can potentially be used to differentiate between impaired baroreflex sensitivity from increased vessel stiffness and other (e.g., neural) causes.

### Baroreflex Sensitivity and Cerebral Autoregulation

BRS reliability was rather low, which may imply that more than three rapid supine to stand repetitions may be necessary to cancel out noise. CAR reliability and validity was higher compared to BRS reliability and validity, which may be explained by the many factors (e.g., emotions, mood, respiration) that influence inter beat interval (Fatisson et al., 2016), which is used to compute BRS, but not CAR.

BRS and CAR showed highest validity when assessed during rapid supine to stand movement, potentially because PPG-ECG-NIRS signal to noise ratio is highest during this postural change.

BRS and CAR computation depends on a good BP estimation, for which an accurate model is necessary. In this study, simple linear regression models were used for to this end to provide first evidence that BP can be estimated from finger PPG signals. However, substantial inter- and intra-individual variation of the regression betas was observed, which implies frequent calibration is necessary to obtain accurate BP estimations. As this is impractical for the goal of continuous estimation of BP, BRS and CAR, more robust models should be developed to estimate BP from PPG-ECG-NIRS data, which may also incorporate heart rate and PWV signals. This could be performed by training neural networks, warranting further research.

Correlations between BRS estimates assessed during postural change and conventional validated BRS measures assessed in rest were rather low, indicating the potential additional value of the BRS estimates assessed during these postural changes. The low correlation may be explained by the non-linear nature of the baroreflex, implying that a BP variation increase with a certain factor is not necessarily followed by a change in inter beat interval variation increase with the same factor (Di Rienzo et al., 2009).

In the present study, BRS was defined as the inter beat interval drop divided by the SBP drop after standing up. The clinical value of this BRS measure needs to be further established by addressing its association with clinical phenotype, e.g., age, presence of orthostatic symptoms, and physical and cognitive performance. Furthermore, the underlying physiology should be elucidated by simultaneous measurements of muscle sympathetic nerve activity (Marchi et al., 2016). A proper functioning baroreflex characterized by inter signal coupling of heart rate, BP and muscle sympathetic nerve activity may be particularly related to clinical phenotype (Barbic et al., 2019). Development of barocontrol models is needed to further disentangle different components contributing to the baroreflex (Porta et al., 2012).

As the NIRS-based measure of CAR used in the present study may apart from cerebral blood flow also be influenced by cerebral microcirculation, further external validation of this CAR measure using cerebral blood flow measurements should be performed in further research, as well as its association with clinical phenotype.

### Accuracy of the BP Measurements Used as a Gold Standard

In this study, continuously and non-invasively measured peripheral BP was used to estimate central (aortic) BP, as in other studies (Pinna et al., 2000; Dell’Oro et al., 2018; Fonkoue et al., 2019; Lázaro et al., 2019; Simpson et al., 2019; Wakeham et al., 2019). Continuously and non-invasively measured peripheral BP was demonstrated to give a good approximation of intra-arterial radial BP in different clinical populations (Pinna et al., 2000; Ameloot et al., 2014; Smolle et al., 2015; Berkelmans et al., 2017).

BP drop after the head up tilt movement was smaller compared BP drop after the active stand movements. This is accordance with results reported in a previous study and may be due to a temporary muscle artery vasodilation during active standing up in contrast to passive tilt, decreasing BP (van Wijnen et al., 2017). This effect counteracts the BP increasing effect of muscle use during standing up through increased venous return and seems to outweigh it in the present and a previous study (van Wijnen et al., 2017).

### Strength and Limitations

The strength of this study is that it systematically assesses the PPG, NIRS, and PWV correlation with BP after postural change and the reliability and validity of the derived BRS and CAR estimates.

The generalizability of this study to the proposed target group of older adults with OH is limited due to the young age of the investigated population. Though the investigated population was relatively healthy, some individuals in the investigated population used drugs affecting the cardiovascular system. Together with the relative underrepresentation of female participants (29.4%), this limits the representativeness of the results for healthy adults. The difference in median age between the subgroups was rather large due to recruitment differences, which limits the comparability of the subgroups. This should be taken into account when comparing the results from two postural changes executed by the two different subgroups, e.g., the rapid supine to stand and supine to stand at preferred speed movements.

The absence of cerebral blood flow measurements as a gold standard for CAR measurements and the fact that 7% of the trials had to be discarded during some repeats due to technical problems or poor signal quality are limitations of this study.

Correlation of finger and wrist PPG with BP was computed while the arm was kept at heart height using a sling, which is a limitation for ambulatory applicability. Future experiments should be independent of this design by correcting PPG based BP estimations for the difference in height between heart and PPG measurement site. This height difference therefore has to be measured separately.

The currently proposed model to estimate BP based on finger PPG is dependent on regular calibration of the data, which is an issue to be addressed in further research.

### Clinical Perspectives and Future Directions

Clinical application of the PPG-ECG-NIRS monitor requires several additional steps, i.e., making the separate sensors entirely wireless, improving the BP estimation algorithms, making BP estimation independent of finger height relative to the heart and further validation of the BRS and CAR estimates in a clinical setting. When these requirements are met, PPG-ECG-NIRS could be applied in patients with impaired mobility or falls, as suspected due to inadequate BP regulation. The PPG-ECG-NIRS monitor should provide a personal risk profile of BP regulation during standing up, which may enable clinicians to personalize treatment, e.g., giving advice on lifestyle changes or revising medication.

Further research should address the association of the investigated parameters with healthy aging and the occurrence of clinical orthostatic symptoms, mobility impairment and falls. It should further address the external validity of the CAR estimates using cerebral blood flow velocity measurements during postural changes. More robust models should be developed to be able to continuously monitor BP, BRS and CAR from PPG-ECG-NIRS signal recordings without the need for frequent calibration.

## Conclusion

PPG and NIRS signals correlated with BP in healthy adults, enabling BP estimation. The BRS and CAR estimates derived from the PPG-ECG-NIRS signals were reliable and valid during supine to stand movements. This study provides evidence of the potential additional value of BRS estimates assessed during postural change compared to conventional validated BRS measures assessed in rest. The results suggest the potential clinical applicability of the PPG-ECG-NIRS signal recordings for continuous unobtrusive monitoring of BP, BRS, and CAR.

## Data Availability Statement

All data and analysis scripts are available via the following link: http://hdl.handle.net/11633/di.dcn.DSC_62002451_01_149.

## Author Contributions

AM, ABM, RW, and CM conceived of the presented idea and designed the study. AM performed the data collection, performed the analysis, and took the lead in writing the manuscript. All authors discussed the results, contributed to the final manuscript, approved the final version of the manuscript and agreed to be accountable for the study, and designated as authors qualify for authorship, and all those who qualify for authorship are listed.

## Conflict of Interest

The authors declare that the research was conducted in the absence of any commercial or financial relationships that could be construed as a potential conflict of interest.
