# Supplementary material for: Multimodal Monitoring of Cardiovascular Responses to Postural Changes
Source: Front Physiol. 2020 Mar 3;11:168. doi: 10.3389/fphys.2020.00168 (PMC7063121; doi:10.3389/fphys.2020.00168)
Supplement: Supplementary file 1 [file Data_Sheet_1.DOCX]

**
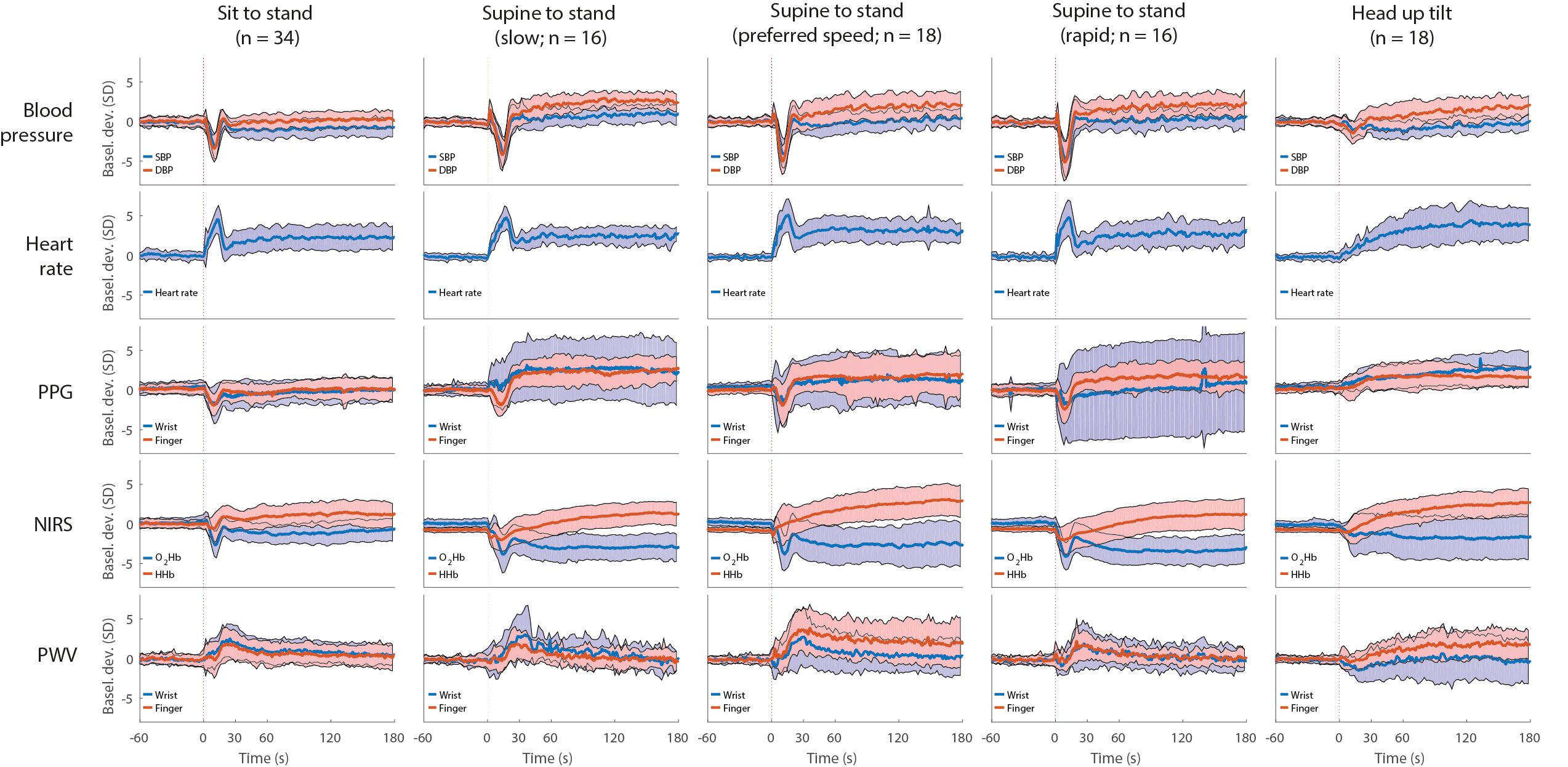
**

**Appendix A: Blood pressure, heart rate, photoplethysmography (PPG), near infrared spectroscopy (NIRS) and pulse wave velocity (PWV) during active and passive postural changes.** All signals are unfiltered and normalized at baseline. The red vertical line indicates the onset of the postural change. The shaded areas indicate the standard deviation. The data represent 24 male subjects and 10 female subjects. Basel. dev.: signal deviation from mean baseline; SBP: systolic blood pressure; DBP: diastolic blood pressure; O_2_Hb: oxygenated hemoglobin; HHb: deoxygenated hemoglobin.

**Appendix B: Signal characteristics during postural changes**

|  | **Postural change** | | | | |
| --- | --- | --- | --- | --- | --- |
|  | **Sit to stand** | **Slow supine to stand** | **Supine to stand at preferred speed** | **Rapid supine to stand** | **Head up tilt** |
|  | **Median [IQR]** | **Median [IQR]** | **Median [IQR]** | **Median [IQR]** | **Median [IQR]** |
| **Mean (0-30s)** | |  |  |  |  |
| SBP, mmHg | -7.7 [-12.2, -4.1] | -8.4 [-11.3, -2.9] | -8.6 [-14.2, -4.3] | -7.7 [-10.8, -3.1] | -2.9 [-7.3, -1.8] |
| DBP, mmHg | -6.2 [-8.3, -3.4] | -4.4 [-7.1, -2.0] | -4.5 [-7.4, -1.5] | -3.8 [-9.8, -0.3] | -1.8 [-4.0, -0.2] |
| O_2_Hb, ∆µmol/L | -1.4 [-2.4, -0.5] | -4.6 [-7.7, -2.0] | -3.6 [-5.9, -1.4] | -5.5 [-8.8, -2.6] | -2.3 [-3.3, -1.1] |
| HHb, ∆µmol/L | 0.0 [-0.1, 0.3] | -0.7 [-1.1, 0.1] | 0.3 [-0.3, 1.1] | -0.7 [-1.7, 0.0] | -0.1 [-0.3, 0.8] |
| PWV wrist, m/s | 0.3 [0.2, 0.5] | 0.3 [0.0, 0.5] | 0.2 [0.0, 0.4] | 0.1 [-0.0, 0.3] | -0.2 [-0.3, -0.1] |
| PWV finger, m/s | 0.1 [0.0, 0.3] | 0.1 [0.0, 0.3] | 0.3 [0.1, 0.5] | 0.2 [-0.0, 0.4] | -0.0 [-0.1, 0.1] |
| **Min (0-30s)** |  |  |  |  |  |
| SBP, mmHg | -21.3 [-31.9, -17.9] | -26.2 [-39.8, -20.2] | -32.8 [-37.9, -23.5] | -30.1 [-39.6, -24.8] | -10.3 [-16.1, -6.6] |
| DBP, mmHg | -18.5 [-22.8, -15.4] | -21.3 [-24.9, -16.0] | -21.8 [-25.2, -19.5] | -24.2 [-27.1, -21.4] | -6.7 [-10.0, -4.6] |
| O_2_Hb, ∆µmol/L | -3.1 [-5.1, -2.4] | -8.3 [-12.0, -5.0] | -5.7 [-10.9, -3.6] | -8.1 [-12.6, -6.4] | -4.1 [-5.8, -2.5] |
| HHb, ∆µmol/L | -0.4 [-0.8, -0.1] | -1.2 [-1.9, -0.7] | -0.4 [-1.1, -0.1] | -1.2 [-2.4, -0.5] | -0.6 [-0.9, -0.2] |
| PWV wrist, m/s | -0.1 [-0.3, 0.0] | -0.4 [-0.7, -0.2] | -0.4 [-0.5, -0.2] | -0.4 [-0.7, -0.3] | -0.5 [-0.6, -0.3] |
| PWV finger, m/s | -0.2 [-0.3, 0.0] | -0.2 [-0.4, -0.1] | -0.1 [-0.3, 0.1] | -0.1 [-0.3, -0.1] | -0.2 [-0.2, -0.1] |
| **Mean (10-60s)** |  |  |  |  |  |
| SBP, mmHg | -7.0 [-9.2, -2.9] | -3.2 [-6.4, 4.9] | -3.8 [-13.2, 0.5] | -2.0 [-4.1, 2.8] | -5.8 [-8.7, -1.3] |
| DBP, mmHg | -1.9 [-4.5, 1.3] | 3.5 [0.5, 7.6] | 2.7 [-1.5, 7.1] | 5.6 [-0.8, 8.1] | 1.1 [-0.7, 4.1] |
| O_2_Hb, ∆µmol/L | -1.7 [-2.6, -0.7] | -4.8 [-9.2, -4.2] | -5.1 [-7.0, -1.5] | -5.7 [-10.2, -3.2] | -3.4 [-5.2, -1.4] |
| HHb, ∆µmol/L | 0.1 [-0.0, 0.5] | 0.0 [-0.7, 0.9] | 0.8 [0.6, 1.5] | -0.2 [-1.1, 0.7] | 0.6 [0.3, 1.5] |
| PWV wrist, m/s | 0.4 [0.3, 0.6] | 0.5 [0.3, 0.7] | 0.5 [-0.1, 0.6] | 0.4 [0.1, 0.7] | -0.2 [-0.3, 0.1] |
| PWV finger, m/s | 0.2 [0.1, 0.3] | 0.2 [0.0, 0.4] | 0.4 [0.2, 0.5] | 0.2 [0.0, 0.6] | 0.0 [-0.0, 0.1] |
| **Max (10-60s)** |  |  |  |  |  |
| SBP, mmHg | 8.2 [0.7, 11.9] | 15.5 [8.5, 18.2] | 9.0 [0.3, 16.3] | 14.3 [7.7, 20.4] | 3.8 [1.7, 6.5] |
| DBP, mmHg | 6.9 [3.2, 9.3] | 15.6 [11.4, 19.6] | 13.3 [10.0, 16.6] | 15.2 [8.5, 18.0] | 9.2 [4.5, 13.9] |
| O_2_Hb, ∆µmol/L | 0.0 [-1.3, 0.8] | -2.4 [-6.3, -0.6] | -2.8 [-5.0, -0.3] | -4.3 [-7.8, -0.8] | -1.3 [-2.3, -0.3] |
| HHb, ∆µmol/L | 0.7 [0.4, 1.0] | 1.1 [0.0, 2.2] | 1.6 [1.4, 2.0] | 0.6 [-0.1, 1.8] | 1.5 [0.8, 2.2] |
| PWV wrist, m/s | 0.8 [0.6, 1.0] | 1.5 [0.8, 1.7] | 0.9 [0.5, 1.0] | 0.9 [0.6, 1.4] | 0.1 [-0.0, 0.5] |
| PWV finger, m/s | 0.5 [0.3, 0.6] | 0.6 [0.3, 0.7] | 0.7 [0.4, 0.9] | 0.5 [0.3, 0.8] | 0.3 [0.2, 0.4] |

Signal characteristics for each postural change. DBP: diastolic blood pressure; IQR: inter quartile range; HHb; deoxygenated hemoglobin; NA: not applicable; O_2_Hb: oxygenated hemoglobin; PPG: photoplethysmography; PWV: pulse transit time; SBP: systolic blood pressure; SD: standard deviation;

**Appendix C: Signal characteristics during the 1-minute squat maneuver**

|  | **Mean (during squat)** | **Mean (60-90s)** | **Min (60-90s)** | **Mean (70-120s)** | |
| --- | --- | --- | --- | --- | --- |
| **SBP [median, IQR]** | 79.5 [70.2, 98.0] | 15.1 [1.1, 17.3] | -7.0 [-18.3, -2.3] | 4.5 [-5.2, 9.1] | |
| **DBP [median, IQR]** | 46.5 [38.9, 58.3] | -3.1 [-4.9, 5.5] | -15.7 [-18.3, -8.7] | -4.8 [-10.0, -3.8] |  |
| **O_2_Hb [median, IQR]** | 16.4 [9.8, 23.1] | 3.1 [1.0, 10.2] | 0.5 [-1.8, 3.2] | 0.8 [-0.5, 3.8] |  |
| **HHb [median, IQR]** | 1.3 [0.9, 1.9] | -1.0 [-1.8, -0.6] | -1.9 [-3.2, -1.4] | -0.7 [-1.5, 0.0] |  |
| **PWV wrist [median, IQR]** | 0.8 [0.6, 1.0] | 0.8 [0.7, 1.2] | -0.0 [-0.1, 0.5] | 1.0 [0.7, 1.1] |  |
| **PWV finger [median, IQR]** | 0.7 [0.4, 1.1] | 0.6 [0.5, 0.8] | 0.1 [-0.0, 0.4] | 0.5 [0.4, 0.6] |  |

Signal characteristics during the 1-minute squat maneuver. DBP: diastolic blood pressure; IQR: inter quartile range; HHb; deoxygenated hemoglobin; NA: not applicable; O_2_Hb: oxygenated hemoglobin; PPG: photoplethysmography; PWV: pulse wave velocity; SBP: systolic blood pressure;
